# Retraction Note: Enhancing residential energy access with optimized stand-alone hybrid solar-diesel-battery systems in Buea, Cameroon

**DOI:** 10.1038/s41598-026-43681-8

**Published:** 2026-03-16

**Authors:** Isaac Amoussou, Eriisa Yiga Paddy, Takele Ferede Agajie, Fuseini Seidu Ibrahim, Elsabet Ferede Agajie, Wirnkar Basil Nsanyuy, Mohit Bajaj, Shir Ahmad Dost Mohammadi

**Affiliations:** 1 Department of Electrical and Electronic Engineering, Faculty of Engineering and Technology, University of Buea, P.O. Box 63, Buea, Cameroon; 2 Department of Electrical and Computer Engineering, Debre Markos University, Debre Markos, Ethiopia; 3 Department of Electrical Engineering, Graphic Era (Deemed to be University), Dehradun, 248002 India; 4 Hourani Center for Applied Scientific Research, Al-Ahliyya Amman University, Amman, Jordan; 5 Graphic Era Hill University, Dehradun, 248002 India; 6 Department of Electrical and Electronics, Faculty of Engineering, Alberoni University, Kohistan, Kapisa Afghanistan

Retraction of: *Scientific Reports* 10.1038/s41598-024-66582-0, published online 05 July 2024

The Editors have retracted this Article.

Following publication, concerns were raised regarding the inclusion of irrelevant references. Further investigation by the Editors revealed inconsistencies between the text and at least 20 of the cited references. In addition, the authorship of the Article could not be verified. Upon request, the Authors could not provide a satisfactory response to the concerns raised.

The Editors therefore no longer have confidence in the reliability of the content of this Article.

Isaac Amoussou and Mohit Bajaj agree to the retraction and its wording. Eriisa Yiga Paddy, Takele Ferede Agajie, Fuseini Seidu Ibrahim, Elsabet Ferede Agajie, Wirnkar Basil Nsanyuy, and Shir Ahmad Dost Mohammadi did not respond to correspondence from the Editors about this retraction.

